# Prevention and management of drug‐induced osteonecrosis of the jaws using platelet‐rich fibrin: A clinical feasibility study

**DOI:** 10.1002/cre2.775

**Published:** 2023-08-21

**Authors:** Abdusalam Alrmali, Muhammad H. A. Saleh, Salaheddin Mohamed S. Kurdi, Hamoun Sabri, Mohamed M. Meghil, Hom‐Lay Wang

**Affiliations:** ^1^ Department of Periodontics and Oral Medicine University of Michigan School of Dentistry Ann Arbor Michigan USA; ^2^ Department of Oral Medicine, Oral Pathology, Oral and Maxillofacial Surgery University of Tripoli School of Dentistry Tripoli Libya; ^3^ Department of Periodontics The Dental College of Georgia at Augusta University Augusta Georgia USA

**Keywords:** autologous platelet concentrate, bisphosphonates, leukocyte—platelet‐rich fibrin, osteonecrosis of the jaw

## Abstract

**Objective:**

Although a standard treatment guideline has not been established to date, various treatment modalities have been described in the literature based on the staging of medication‐related osteonecrosis of the jaw (MRONJ). The aim of this case series was to describe the outcomes of surgical intervention of MRONJ cases with the adjunctive use of platelet‐rich fibrin (PRF).

**Materials and Methods:**

Thirteen patients under therapy with zoledronic acid, seven of them underwent surgical removal of necrotic bone with debridement, followed by placement of three to four PRF membranes and achieving primary closure. In six patients, PRF was used preventively to avoid MRONJ.

**Results:**

The surgical treatment outcomes were successful in all patients, with a follow‐up range of 12–48 months. In the presented cases, the intraoral evaluation showed excellent soft tissue healing except for one patient secondary wound healing was reported. Additionally, there was no recurrence of bone exposure in all cases. PRF membranes were comparatively effective in postsurgical pain control.

**Conclusion:**

The use of PRF could represent a valuable adjunct in the surgical management for advanced stages of MRONJ cases.

**Clinical Relevance:**

This clinical case series describes the use of PRF membranes as a valuable adjunct in the surgical management of MRONJ patients, especially when treating advanced MRONJ cases. Moreover, PRF demonstrates usefulness in preventing such difficult complications from occurring.

## 1 INTRODUCTION

Medication‐related osteonecrosis of the jaw (MRONJ) was first described by Marx in 2003 as a complication among cancer or osteoporosis patients who had received bisphosphonate (BP) therapy with no previous history of radiation therapy of the jaw (Marx et al., 2005; Ruggiero et al., 2014). Ever since, the literature formerly describing this condition has constantly grown with regard to BPs and several other medication families such as denosumab (DMB) for which several treatment protocols have been proposed (ICD‐10; Ruggiero et al., 2014, 2022). As the main effect of the BPs is on the osteoclasts, they interfere with the process of bone turnover by inhibiting the resorption of trabecular bone, hence increasing bone density, and impairing angiogenesis (Marx et al., 2005; Ruggiero et al., 2014; ICD‐10). The main indication for BPs is to reduce the risk of fractures in patients with osteoporosis/osteopenia and to decrease the risk of metastasis in cancer patients, especially breast and prostate cancers (Ruggiero et al., 2014, 2022). One of the consequences of impaired bone healing and turnover in the jaws, particularly following common oral surgical procedures such as tooth extraction or implant placement, is the exposure of the bone to the oral environment. This can lead to the development of infection, pain, and even jawbone fracture (Marx et al., 2005). Generally, the greatest initiator of MRONJ is a tooth extraction (Advisory Task Force on Bisphosphonate‐Related Ostenonecrosis of the Jaws, American Association of Oral and Maxillofacial Surgeons, 2007).

Other classes of non‐BP drugs (mainly antiresorptive or antiangiogenic medications) are associated with clinical features of bone exposure in the jaws (Advisory Task Force on Bisphosphonate‐Related Ostenonecrosis of the Jaws, American Association of Oral and Maxillofacial Surgeons, 2007; Ruggiero et al., 2022; Sarkarat et al., 2022). “In the recent Position Paper of the American Association of Oral and Maxillofacial Surgeons (AAOMS) published in 2022 (Ruggiero et al., 2022), the term medication‐related osteonecrosis of the jaw (MRONJ) remains unchanged,” where the World Health Organization coined the term drug‐induced osteonecrosis of the jaws (DIONJ) (ICD‐10). In the last update of the same article in 2022, the lesions of MRONJ still staged into stages 0, 1, 2, and 3 (Ruggiero et al., 2022). In the first stage, the patient is asymptomatic with exposed and necrotic bone or fistula that probes to the bone without evidence of infection or inflammation. These patients also may present with radiographic findings mentioned for stage 0 that are localized to the alveolar bone region. The final stage, stage 3, is characterized by exposed necrotic bone in patients experiencing pain, infection, and complications such as pathologic fracture, extraoral fistula, and oral antral/oral nasal communication (Ruggiero et al., 2022).

Currently, there is no standardized protocol for treating MRONJ cases (Ruggiero et al., 2022). However, many treatment modalities have been described in the literature depending on the stage and degree of bone necrosis (Alrmali, 2018; Sarkarat et al., 2014). Over the years, a conservative approach has been the first choice of treatment including local debridement, removal of bone sequestrum, and a combination of systemic antibiotic treatment and/or antiseptic solutions (e.g., chlorhexidine) (Myneni Venkatasatya et al., 2017; Ruggiero et al., 2022). These yield superior outcomes in the early stages of MRONJ (Ruggiero et al., 2022). In the majority of the cases, a reduction in the clinical signs and symptoms or complete healing can be achieved (Ruggiero et al., 2022). In recent years, some therapeutic protocols have been proposed to enhance the healing and the predictability of the surgical treatment, such as ozone therapy (Ripamonti et al., 2011), low‐level laser therapy (Sarkarat et al., 2019; Scoletta et al., 2010), hyperbaric oxygen (Freiberger et al., 2012), autologous platelet concentrates (APCs) (Adornato et al., 2007; Del Fabbro et al., 2018; Miranda et al., 2021; Sarkarat et al., 2014) and combination therapies (Sarkarat et al., 2022). The combination of the surgical treatment and autologous membranes of platelet‐rich fibrin (PRF) has the objective of adding bioactive factors to the surgical site to accelerate bone and soft tissue healing (Adornato et al., 2007; Mohammadi et al., 2022; Nørholt & Hartlev, 2016; Sabri et al., 2022). Recent systematic reviews on this topic have concluded that there is still insufficient evidence to prove the true benefits of platelet concentrates in the healing of ONJ lesions (Fabbro et al. 2015, 2018; Fortunato et al., 2020; Lopez‐Jornet et al., 2016). Therefore, this study aimed to propose a protocol for the management and prevention of MRONJ using PRF and describe the treatment outcomes in a series of patients.

## 2 CASE SERIES

### 2.1 Study design and subject recruitment

This study was designed as a prospective clinical feasibility report. The procedures employed were in accordance with the ethical standards of the 1975 Declaration of Helsinki revised in 2013. All the patients underwent surgical treatment at a private practice setting (between 2016 and 2021) at Tripoli and Misurata cities, Libya. Ethical approval was obtained from the Ethics Committee, Ministry of health, registration number: 00002A/16. Signed informed consent was obtained from all patients. The surgical intervention was performed by one surgeon (A. A). The study was also conducted in conformance with strengthening the reporting of observational studies in epidemiology guidelines: http://www.strobe-statement.org (Vandenbroucke et al., 2007). The inclusion criteria were (1) diagnosis of MRONJ and need for surgical management either under local or general anesthesia or patients under the risk of developing MRONJ and requiring preventive intervention; (2) patients able to undergo surgical treatment (American Society of Anesthesiologists‐2 [ASA‐2] or ASA‐3); and the exclusion criteria was reserved for: (1) any contraindications or conditions limiting the surgical intervention. The following information was collected: demographic data, medication (antiresorptive drug or antiangiogenic), duration of the therapy (time of BPs use), route of administration (oral or intravenous [IV]), location of the lesion, surgical site, MRONJ stage, number of PRF membranes utilized, and postoperative follow‐up.

### 2.2 Diagnosis

The diagnosis was made based on the diagnostic criteria of MRONJ, according to the position article published in 2022 (Ruggiero et al., 2022). This diagnosis was confirmed in all patients after clinical examination, which included a review of their past medical and dental history, as well as oral and radiographic examinations.

### 2.3 Treatment and management protocol

#### 2.3.1 General treatment protocol

Oral antibiotic treatment with a combination of amoxicillin and clavulanic acid were initiated the day before surgery (Clavulin BD1, 875/125 mg; GlaxoSmithKline) and continued for every 12 h for 9 more days.

**Figure 1:**
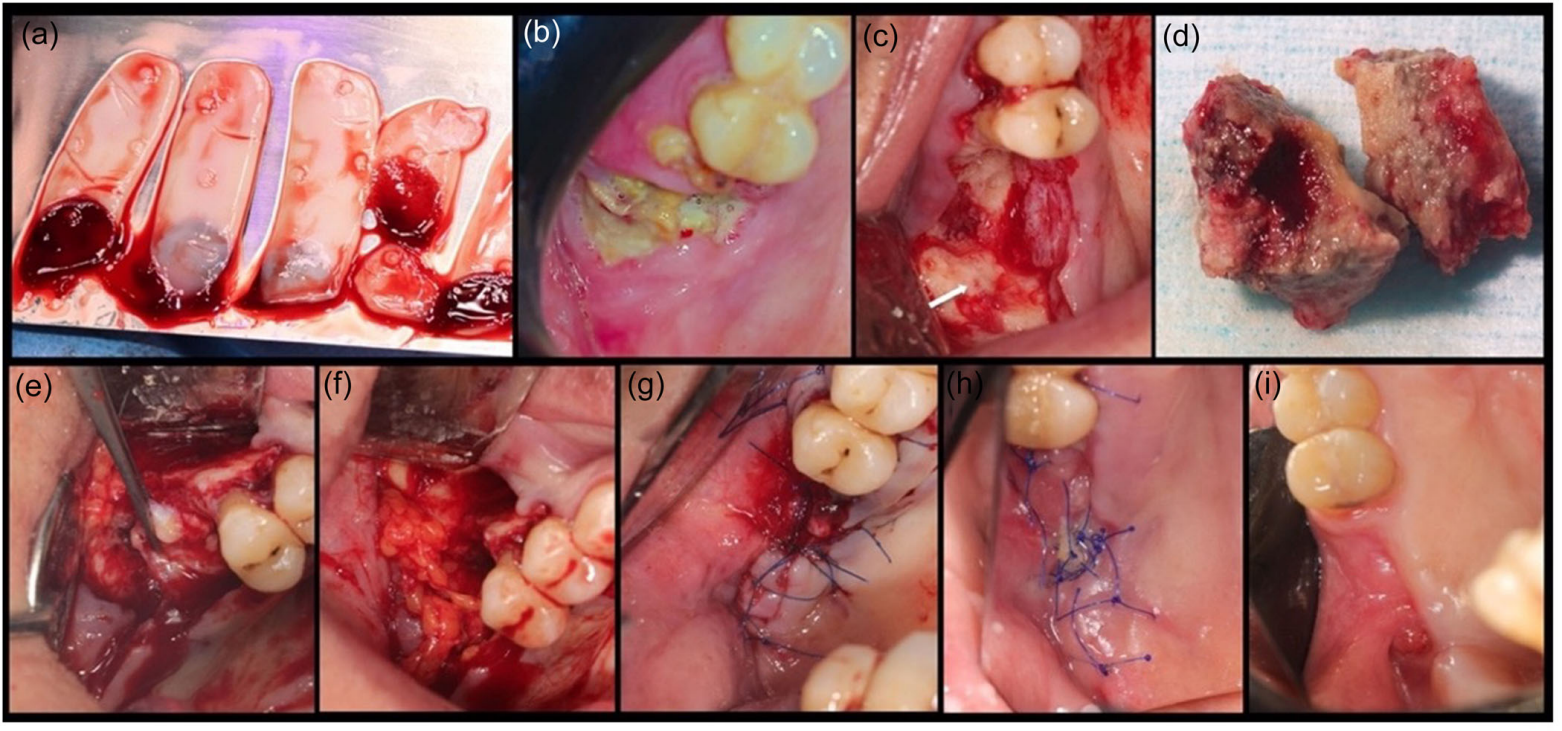
(a) Platelet‐rich fibrin (PRF) membranes prepared; (b) preoperative photos of idiopathic medication‐related osteonecrosis of the jaw of the maxilla; (c) resection of the necrotic bone and decortication of the maxillary sinus floor; (d) removal of the necrotic bone; (e, f) application of buccal fat pad over the PRF and closure using a palatal pedicled flap; (g) primary closure achieved; (h) 2‐week healing; (i) 8‐week healing.

**Figure 2:**
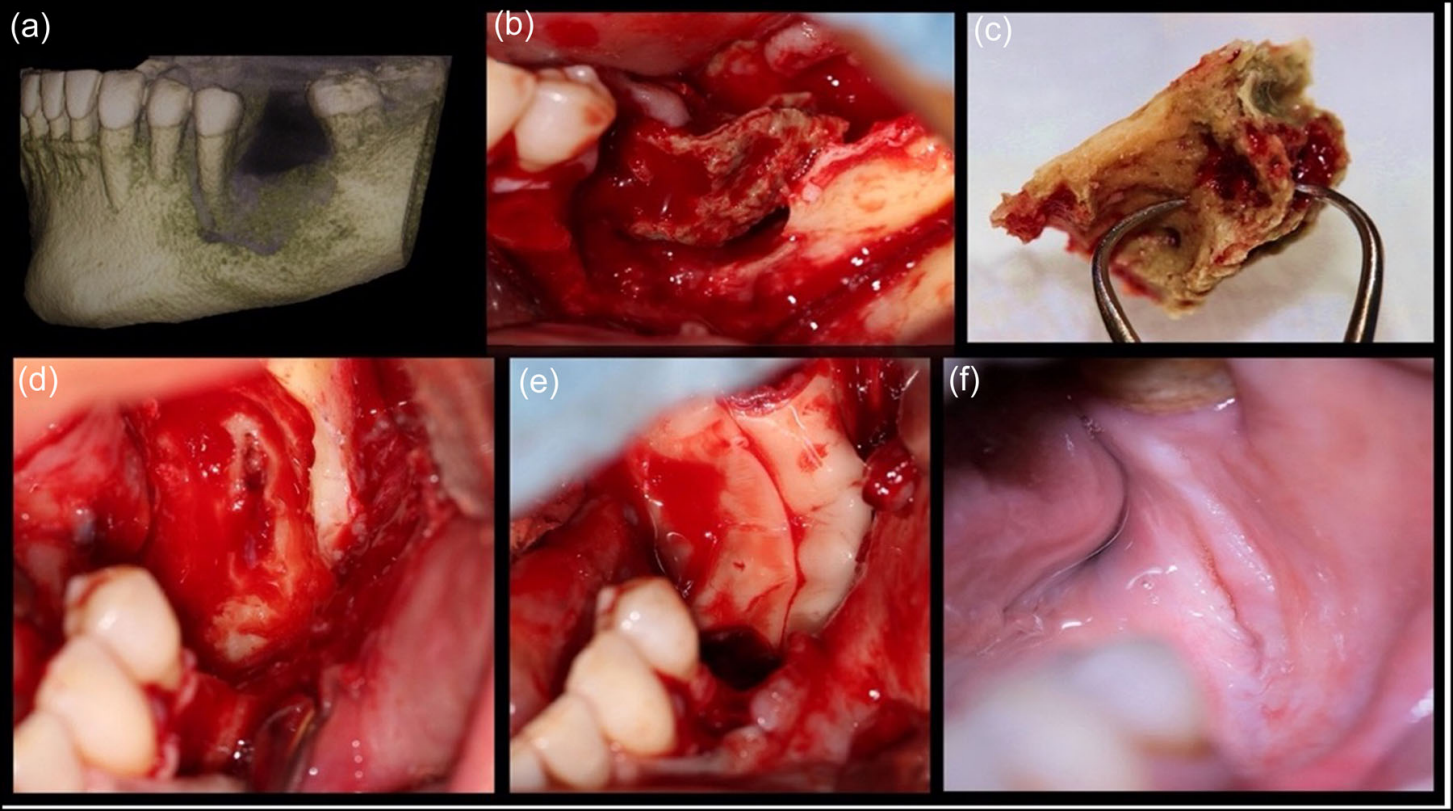
(a) Cone beam computed tomography image showing the boney sequestrum; (b) the necrotic bone was readily removal following flap elevation; (c) removal of the necrotic bone; (d) decortication of the underlying bone was performed following complete removal of the necrotic bone; (e) application of the platelet‐rich fibrin into the defect area; (f) 12‐week healing.

**Figure 3:**
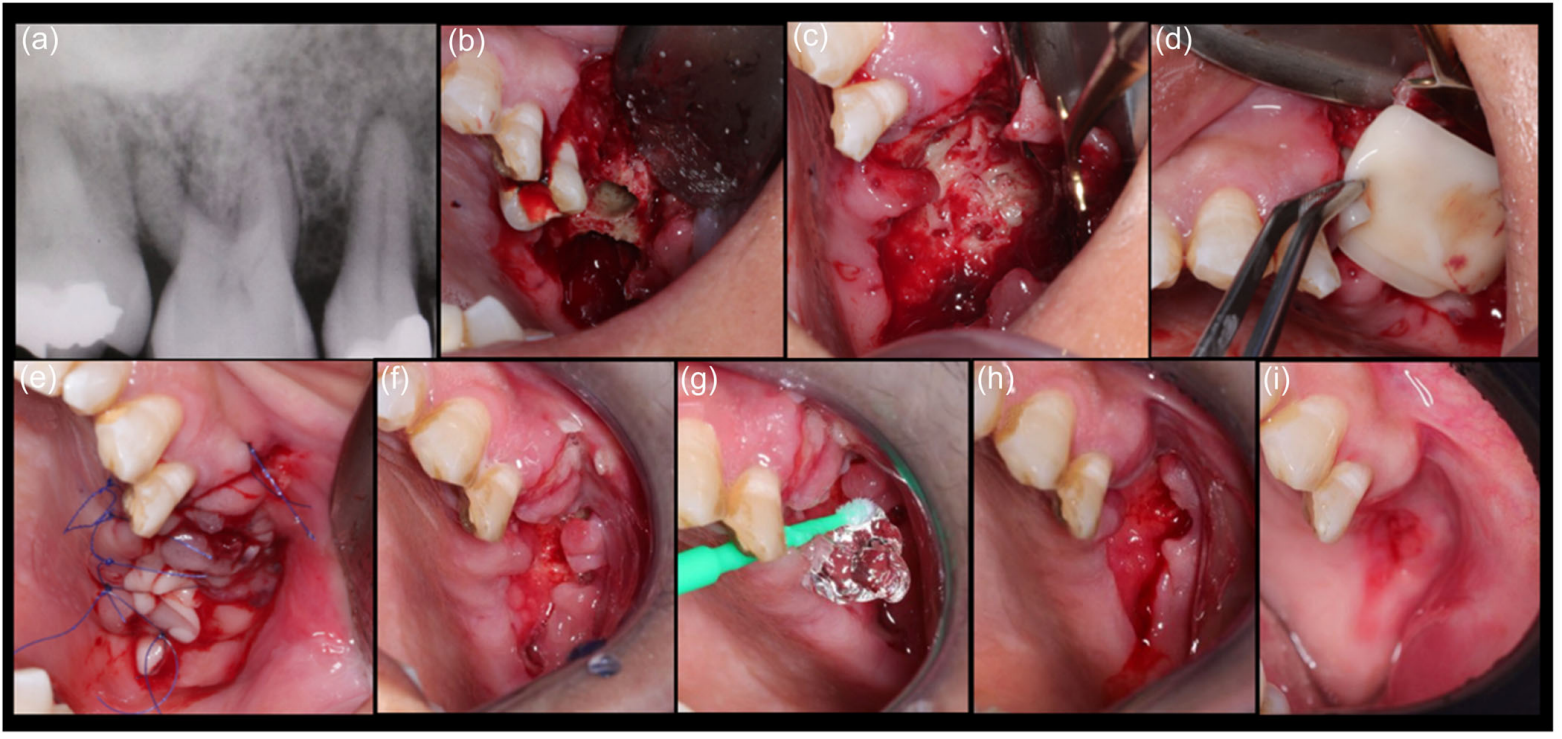
(a) Periapical radiograph image showing changes in trabecular pattern; (b) the necrotic bone was involved inter radicular and buccal bones of the upper first molar socket; (c) removal of the necrotic bone and decortication of the maxillary sinus floor was performed; (d) application of the PRF into the defect area in layers; (e) primary closure achieved; (f) 2‐week healing; (g) application of 1% hyaluronic acid over the wound; (h) 8‐week healing; (i) 24‐week healing.

All the patients had been receiving oral corticosteroids all of whom continued the use of their medication, according to the recommendation by their medical doctor. After proper surgical preparation, medical consultation was done. Standard surgical protocol was followed for all patients to eradicate the defects under aseptic technique and with the concept of applying PRF into the defected areas further followed by applying a chlorhexidine 0.2% + hyaluronic acid 1% gel (PerioKIN, Kin Laboratories, S. A.) (Figures 1, 2, and 3d,g).

#### 2.3.2 PRF preparation protocol

Before starting the surgery, blood samples were collected from each patient with a 21‐gauge vein needle. Six to eight collection tubes of 10‐mL were filled, without the addition of anticoagulants. After collection, the blood was immediately centrifuged with a force of approximately 400*g* (2700 rpm) for 12 min.^1^ After centrifugation, the tubes were placed vertically in a rack, allowing for the blood to clot for almost 15–20 min. At the end of the procedure, the clot (PRF) was removed from the collection tubes bluntly and placed into the PRF box, a light pressure was applied to the PRF clot with PRF‐Box to form a membrane^2^ (Figure 1a).

#### 2.3.3 Surgical intervention

Seven patients were treated under local anesthesia with 2% lidocaine containing 1:100,000 epinephrine. While two of them were admitted to the hospital for general anesthesia due to the severity of the necrosis that could not be treated under local anesthesia. To access the surgical site, a mucoperiosteal flap was extended from mesial to the distal of the lesion, and it was elevated and mobilized by performing deep split and superficial split to facilitate tension‐free primary closure. Necrotic bone was removed with surgical bur and the bone surface underwent surgical debridement to remove all necrotic bones (Figure 1a–i). The extent of the resection was based on the preoperative cone beam computed tomography (CBCT) findings and intraoperative appearance of the bone vitality (bleeding) at the resected surface. The first key after removal of the necrotic bone is to identify the healthy bone by creating small holes using a small round bur under copious irrigation to improve the blood supply to the area and identify bone vitality. Any sharp edges were smoothened and the residual bone was perforated using a small round bur to enhance the blood supply. After this, flap release by periosteal scoring was done to achieve primary closure. The amount of PRF membranes to use was left to the surgeon's decision and it varied for each case depending on the extension of the surgical bone defect (Table 1). In all cases, PRF was placed in layers directly over the perforated bone. Finally, the flap was sutured with 5‐0 polypropylene sutures,^3^ and this would be the second key as primary closure is critical in such cases (Figure 2a–f). Postoperative instructions and medications were given, and careful follow‐up was done. A soft diet was prescribed for 4 weeks, and topical 0.2% chlorhexidine (Periodex) was used for 2 weeks after the surgical procedure. This was continued with 0.012% for another 2 weeks. The patients were strictly followed every week for the first month until achieving soft tissue closure and elimination of any symptoms. Next, they were scheduled for follow‐up visits (Figure 3a–i).

**Table 1:** Summarizes demographic data, treatment variables, and clinical outcomes for patients with different MRONJ stages treated using surgical intervention with PRF membranes.

Case no	Aim	Age	Sex	Medications and route of administration	Time of use	Stage according to AAOMS 2022	Location	Occurrence	PRF tubes	Follow‐up (months)
1	Treatment	43	F	IV zolendronic acid	2 years	Stage 3	Posterior maxilla	History of extraction	6	36
2	Treatment	74	F	Oral alendronate	7 years	Stage 3	Posterior mandible	Spontaneous	8	48
3	Treatment	68	F	IV zolendronic acid	4 years	Stage 2	Posterior mandible	History of extraction	6	12
4	Treatment	67	F	IV zolendronic acid	1 year	Stage 2	Anterior maxilla	History of extraction	6	12
5	Treatment	71	M	IV zolendronic acid	3 years	Stage 2	Anterior mandible	History of extraction	4	12
6	Treatment	68	M	IV zolendronic acid	2 years	Stage 3	Posterior maxilla	Spontaneous	8	18
7	Treatment	54	F	IV zolendronic acid	3 years	Stage 3	Posterior maxilla	Spontaneous	8	12
8	Prevention	43	F	IV zolendronic acid	More than 1 year and current	‐	Posterior mandible	‐	4	36
9	Prevention	74	F	Oral alendronate	7 years	‐	Anterior mandible	‐	4	48
10	Prevention	44	F	IV zolendronic acid	More than 1 year and current	‐	Posterior maxilla	‐	4	12
11	Prevention	48	F	IV zolendronic acid	More than 1 year and current	‐	Posterior maxilla	‐	4	24
12	Prevention	45	F	IV zolendronic acid	More than 1 year and current	‐	Posterior maxilla	‐	4	12
13	Prevention	62	F	Oral alendronate	More than 3 years and current	‐	Posterior mandible	‐	4	24

Abbreviations: AAOMS, American Association of Oral and Maxillofacial Surgeons; F, female; IV, intravenous; M, male; MRONJ, medication‐related osteonecrosis of the jaw; PRF, platelet‐rich fibrin.

### 2.4 Prevention protocol

To prevent the reoccurrence or occurrence of MRONJ in patients who are at risk, a protocol based on the experience of the authors using PRF is proposed as follows. Six preventive cases were included in the study, two of whom had a history of MRONJ and were treated among the aforementioned treatment group. Based on the evaluation, two patients were under therapy with oral alendronate for osteoporosis, and four were receiving IV zolendronic acid for the treatment of breast cancer metastasis. The patients did not report any previous treatment in the affected area. The same steps of clinical examination, including history, oral, and radiographical examinations, were performed for the tooth which was indicated for extraction. Overall, for the preventive cases, atraumatic extraction was carried out. Next, multiple PRF plugs were condensed in the socket with multiple cross sutures without achieving primary closure.

## 3 RESULTS AND CLINICAL OUTCOMES

Overall, 13 patients (11 females and 2 males) were included to this study. The mean age of the subjects was 58 ± 12 years (range 43–74 years). The complete demographic data of the patients is depicted in Table 1. The number of affected mandibles and maxillae were almost equal among the patients; four cases in maxilla versus three cases in mandible. MRONJ was triggered after a tooth extraction in four patients. While in three patients it had occurred spontaneously. Two of the patients (#2 and #13) had been taking 70 mg tablet of alendronate sodium orally per week. The mean duration of medication therapy before the onset of MRONJ was 3.14 ± 1.8 years (range 36–84 months). The outcome of the surgical treatment was successful in all patients (100%) except in one case patient #5 in which flap dehiscence occurred. This case kept under mouthwash use until secondary wound granulation and disappearance of any symptoms. The mean follow‐up period was 24.5 ± 14.11 months (range 12–48 months). The clinical evaluation showed excellent soft tissue healing at any follow‐up appointments, without bone exposure and signs of infections. In most cases, complete soft tissue closure was achieved by 2–4 weeks. Local pain disappeared within the first week postsurgery in all patients. No complications occurred throughout the follow‐up period except for one case. No recurrence was reported among all the cases during follow‐up period.

## 4 DISCUSSION

MRONJ causes significant morbidity and adversely affects the patient's quality of life. Several strategies and treatment modalities for management of patients with or at risk of MRONJ have been proposed in previous clinical studies and consensus (Ruggiero et al., 2014, 2022). The current paper presents the treatment and prevention of MRONJ in 13 patients, 12 females, and 1 male, using PRF. The higher incidence rate of MRONJ in females has been reported in previous studies. Furthermore, spontaneous MRONJ occurs four times more commonly in females than males (Urade et al., 2011). In addition, three patients had a history of taking the oral BP (alendronate) while 10 patients had been receiving IV BP (Zoledronic acid). Consistently, a 4‐year prospective study by “Hallmer et al. (2018), showed that MRONJ occurs more frequently in patients taking IV bisphosphonates than oral bisphosphonates.” While patients with IV BPs have increased risk of developing MRONJ following dental extraction (Khominsky & Lim, 2018), some clinical reports also suggest a spontaneous occurrence of MRONJ in this population of patients (Wysowski & Greene, 2013). Conversely, other studies reported more frequent incidence of MRONJ in patients taking oral BPs, attributed to the high prescription rates of oral BPs (Vandone et al., 2012).

Many clinical strategies addressing the treatment and prevention of MRONJ. These proposed strategies were tested and shown to enhance healing and reduce the risk of MRONJ in patients on BPs (Ferlito et al., 2011). BPs inhibit osteoclastic function by interfering with osteoclast recruitment, differentiation, and resorptive activity. Considering albeit a drug holiday can reverse their systemic effect, it is unlikely to affect the activity of bone matrix‐bound BPs (Ruggiero et al., 2022). However, the use of antibiotics before and/or after the surgical procedure (Schubert et al., 2012), the use of antimicrobial mouthwashes (Lodi et al., 2010), appropriate closure of the wound following tooth extraction (Francini et al., 2011), good oral hygiene (Weiss et al., 2008), the use of neodymium‐doped yttrium aluminium garnet laser (Vescovi et al., 2007), and the use of growth factors are all prove helpful antibacterial properties and in minimizing the risks of MRONJ (Asaka et al., 2017; Fabbro et al., 2016; Feng et al., 2020; Fernando de Almeida Barros Mourão et al., 2020; Yüce et al., 2021).

Furthermore, our current study involved seven cases of MRONJ that were treated with debridement of necrotic bone and application of PRF. Whereas for the other six cases who had been taking BPs, PRF was used to prevent the occurrence of MRONJ. The average duration of BPs therapy for those patients was 3.14 ± 1.8 years. All cases healed properly with neither complications nor recurrence of MRONJ postsurgically. The concept of employing platelet‐derived plasma as a therapeutic agent in the treatment of MRONJ was tested in a split‐mouth animal study by “Sarkarat et al. (2014), who showed that a significantly higher sound bone formation in the PRP group than the non‐PRF treated groups in zoledronic acid induced MRONJ defects.” This is because PRF induces the survival and proliferation of fibroblast and keratinocytes, and PRF also antagonizing the effects of BPs as well as possess antibacterial function. In addition, PRF was used by others to prevent MRONJ due to the high concentration of platelet‐derived growth factors (PDGF), which plays a crucial role as an essential regulator for the migration, proliferation, and survival of mesenchymal cells. In addition, PRF contains transforming growth factor‐β1, a growth factor that is released by autologous bone, which stimulates synthesis of type 1 collagen and fibronectin and vascular endothelial growth factor (VEGF), the most potent growth factor leading to tissue angiogenesis (Mohammadi et al., 2022).

When it comes to using autogenous platelet concentrates as preventive agents before extractions, the results of a systematic review by “Fortunato et al. (2020) indicated that there is still lack of sufficient evidence to conclude its efficacy.” In addition, it was not mentioned in the last position paper updated protocol (Ruggiero et al., 2022). However, several other recent human studies queried the feasibility of the use of PRF as a preventive strategy for the patients taking BPs and reported promising results. “Miranda et al. (2021), in a comparative study design, compared occurrence of MRONJ after the extraction and found the PRF group showed no occurrence while in the no PRF group an approximately 20% of the patients developed MRONJ.” Based upon the findings from literature and the reported results, this case series strengthen and support the concept of using PRF in the management of MRONJ cases or the patients under high risk of developing it.

Readers should be borne in mind that due to the case‐series nature of this study; the results should be interpretated with cautious. Future studies with larger sample sized and the longer follow‐up are needed to validate the results of this clinical feasibility study.

Within its limitations, the results of this case series indicated that the use of PRF could represent a valuable adjunct in the treatment and prevention of MRONJ due to its biological action attributed to high concentrations of growth factors that increase angiogenesis to enhance bone repair and promote soft tissue healing to achieve primary wound closure.

## AUTHOR CONTRIBUTIONS

*Conceptualization*: Abdusalam Alrmali and Salaheddin Mohamed S. Kurdi. *Methodology*: Abdusalam Alrmali, Mohamed M. Meghil, and Muhammad H. A. Saleh. *Software*: None. *Validation*: Abdusalam Alrmali, Hamoun Sabri, and Hom‐Lay Wang. *Formal Analysis*: Abdusalam Alrmali. *Investigation*: Abdusalam Alrmali. *Resources*: Salaheddin Mohamed S. Kurdi. *Data Curation*: Abdusalam Alrmali. *Writing—Original Draft Preparation*: Abdusalam Alrmali and Muhammad H. A. Saleh. *Writing—Review and Editing*: Hamoun Sabri and Mohamed M. Meghil. *Visualization*: Muhammad H. A. Saleh. *Supervision*: Hom‐Lay Wang. *Project Administration*: Abdusalam Alrmali. *Funding Acquisition*: None. All authors gave their final approval of the version to be published and accountable to the accuracy or integrity of the work.

## CONFLICT OF INTEREST STATEMENT

The authors declare no conflict of interest.

## ETHICS STATEMENT

Ethical approval was obtained from the Ethics Committee, Ministry of health, registration number: 00002A/16. All procedures performed in studies involving human participants were in accordance with the ethical standards of the institutional and/or national research committee and with the 1975 Helsinki Declaration revised in 2013. Informed consent was obtained from all individual participants included in the study.

## Data Availability

The data that support the findings of this study are available from the corresponding author upon reasonable request.

## Footnotes

1 Intra‐Spin1 EBA 200, BioHorizons.

2 Intra‐Lock System, BioHorizon.

3 Maxon™▫ 5/0 monofilament absorbable sutures, Medtronic.

## References

[cre2775-bib-0001] Adornato, M. C. , Morcos, I. , & Rozanski, J. (2007). The treatment of bisphosphonate‐associated osteonecrosis of the jaws with bone resection and autologous platelet‐derived growth factors. The Journal of the American Dental Association, 138, 971–977. 10.14219/jada.archive.2007.0294 17606496

[cre2775-bib-0002] Advisory Task Force on Bisphosphonate‐Related Ostenonecrosis of the Jaws, American Association of Oral and Maxillofacial Surgeons . (2007). American Association of Oral and Maxillofacial Surgeons position paper on bisphosphonate‐related osteonecrosis of the jaws. Journal of Oral and Maxillofacial Surgery, 65, 369–376. 10.1016/j.joms.2006.11.003 17307580

[cre2775-bib-0003] Alrmali, A. (2018). A minimally invasive protocol for the prevention and management of medication‐related osteonecrosis of the jaw. Clinical Oral Implants Research, 29, 465.29569763

[cre2775-bib-0004] Asaka, T. , Ohga, N. , Yamazaki, Y. , Sato, J. , Satoh, C. , & Kitagawa, Y. (2017). Platelet‐rich fibrin may reduce the risk of delayed recovery in tooth‐extracted patients undergoing oral bisphosphonate therapy: A trial study. Clinical Oral Investigations, 21, 2165–2172. 10.1007/s00784-016-2004-z 27837344

[cre2775-bib-0005] Del Fabbro, M. , Gallesio, G. , & Mozzati, M. (2015). Autologous platelet concentrates for bisphosphonate‐related osteonecrosis of the jaw treatment and prevention. A systematic review of the literature. European Journal of Cancer, 51, 62–74. 10.1016/j.ejca.2014.10.015 25466505

[cre2775-bib-0006] Del Fabbro, M. , Taschieri, S. , & Goker, F. (2018). Platelet concentrates as an adjunctive therapy for medication‐related osteonecrosis of the jaw: A systematic review and meta‐analysis. International Journal of Growth Factors and Stem Cells in Dentistry, 1, 48.

[cre2775-bib-0007] Fabbro, M. D. , Bortolin, M. , Taschieri, S. , Ceci, C. , & Weinstein, R. L. (2016). Antimicrobial properties of platelet‐rich preparations. A systematic review of the current pre‐clinical evidence. Platelets, 27, 276–285. 10.3109/09537104.2015.1116686 26763769

[cre2775-bib-0008] Feng, M. , Wang, Y. , Zhang, P. , Zhao, Q. , Yu, S. , Shen, K. , Miron, R. J. , & Zhang, Y. (2020). Antibacterial effects of platelet‐rich fibrin produced by horizontal centrifugation. International Journal of Oral Science, 12, 32. 10.1038/s41368-020-00099-w 33243983 PMC7693325

[cre2775-bib-0009] Ferlito, S. , Puzzo, S. , & Liardo, C. (2011). Preventive protocol for tooth extractions in patients treated with zoledronate: A case series. Journal of Oral and Maxillofacial Surgery, 69, e1–e4. 10.1016/j.joms.2010.10.055 21316136

[cre2775-bib-0010] Fernando de Almeida Barros Mourão, C. , Calasans‐Maia, M. D. , Del Fabbro, M. , Le Drapper Vieira, F. , Coutinho de Mello Machado, R. , Capella, R. , Miron, R. J. , & Gomes Alves, G. (2020). The use of platelet‐rich fibrin in the management of medication‐related osteonecrosis of the jaw: A case series. Journal of Stomatology, Oral and Maxillofacial Surgery, 121, 84–89. 10.1016/j.jormas.2019.02.011 30794883

[cre2775-bib-0011] Fortunato, L. , Bennardo, F. , Buffone, C. , & Giudice, A. (2020). Is the application of platelet concentrates effective in the prevention and treatment of medication‐related osteonecrosis of the jaw? A systematic review. Journal of Cranio‐Maxillofacial Surgery, 48, 268–285. 10.1016/j.jcms.2020.01.014 32063481

[cre2775-bib-0012] Francini, F. , Pascucci, A. , Francini, E. , Miano, S. T. , Bargagli, G. , Ruggiero, G. , & Petrioli, R. (2011). Osteonecrosis of the jaw in patients with cancer who received zoledronic acid and bevacizumab. The Journal of the American Dental Association, 142, 506–513. 10.14219/jada.archive.2011.0220 21531932

[cre2775-bib-0013] Freiberger, J. J. , Padilla‐Burgos, R. , McGraw, T. , Suliman, H. B. , Kraft, K. H. , Stolp, B. W. , Moon, R. E. , & Piantadosi, C. A. (2012). What is the role of hyperbaric oxygen in the management of bisphosphonate‐related osteonecrosis of the jaw: A randomized controlled trial of hyperbaric oxygen as an adjunct to surgery and antibiotics. Journal of Oral and Maxillofacial Surgery, 70, 1573–1583. 10.1016/j.joms.2012.04.001 22698292

[cre2775-bib-0014] Hallmer, F. , Andersson, G. , Götrick, B. , Warfvinge, G. , Anderud, J. , & Bjørnland, T. (2018). Prevalence, initiating factor, and treatment outcome of medication‐related osteonecrosis of the jaw‐a 4‐year prospective study. Oral Surgery, Oral Medicine, Oral Pathology and Oral Radiology, 126, 477–485. 10.1016/j.oooo.2018.08.015 30249535

[cre2775-bib-0015] ICD‐10 . *International statistical classification of diseases and related health problems, 10th revision*. Retrieved October 2022 from, https://icd.who.int/browse10/2019/en

[cre2775-bib-0016] Khominsky, A. , & Lim, M. (2018). “Spontaneous” medication‐related osteonecrosis of the jaw; two case reports and a systematic review. Australian Dental Journal, 63, 441–454. 10.1111/adj.12648 30144095

[cre2775-bib-0017] Lodi, G. , Sardella, A. , Salis, A. , Demarosi, F. , Tarozzi, M. , & Carrassi, A. (2010). Tooth extraction in patients taking intravenous bisphosphonates: A preventive protocol and case series. Journal of Oral and Maxillofacial Surgery, 68, 107–110. 10.1016/j.joms.2009.07.068 20006163

[cre2775-bib-0018] Lopez‐Jornet, P. , Sanchez Perez, A. , Amaral Mendes, R. , & Tobias, A. (2016). Medication‐related osteonecrosis of the jaw: Is autologous platelet concentrate application effective for prevention and treatment? A systematic review. Journal of Cranio‐Maxillofacial Surgery, 44, 1067–1072. 10.1016/j.jcms.2016.05.004 27318752

[cre2775-bib-0019] Marx, R. E. , Sawatari, Y. , Fortin, M. , & Broumand, V. (2005). Bisphosphonate‐induced exposed bone (osteonecrosis/osteopetrosis) of the jaws: Risk factors, recognition, prevention, and treatment. Journal of Oral and Maxillofacial Surgery, 63, 1567–1575. 10.1016/j.joms.2005.07.010 16243172

[cre2775-bib-0020] Miranda, M. , Gianfreda, F. , Raffone, C. , Antonacci, D. , Pistilli, V. , & Bollero, P. (2021). The role of platelet‐rich fibrin (PRF) in the prevention of medication‐related osteonecrosis of the jaw (MRONJ). BioMed Research International, 2021, 1–8. 10.1155/2021/4948139 34095295 PMC8140838

[cre2775-bib-0021] Mohammadi, A. , Dehkordi, N. R. , Mahmoudi, S. , Rafeie, N. , Sabri, H. , Valizadeh, M. , Poorsoleiman, T. , Jafari, A. , Mokhtari, A. , Khanjarani, A. , Salimi, Y. , Mokhtari, M. , & Deravi, N. (2022). Effects of drugs and chemotherapeutic agents on dental implant osseointegration: Narrative review. Current Reviews in Clinical and Experimental Pharmacology, 17, 42–60. 10.2174/2772432817666220607114559 35674294

[cre2775-bib-0022] Myneni Venkatasatya, S. R. , Wang, H. H. , Alluri, S. , & Ciancio, S. G. (2017). Phosphate buffer‐stabilized 0.1% chlorine dioxide oral rinse for managing medication‐related osteonecrosis of the jaw. American Journal of Dentistry, 30, 350–352. 29251459

[cre2775-bib-0023] Nørholt, S. E. , & Hartlev, J. (2016). Surgical treatment of osteonecrosis of the jaw with the use of platelet‐rich fibrin: A prospective study of 15 patients. International Journal of Oral and Maxillofacial Surgery, 45, 1256–1260. 10.1016/j.ijom.2016.04.010 27179556

[cre2775-bib-0024] Ripamonti, C. I. , Cislaghi, E. , Mariani, L. , & Maniezzo, M. (2011). Efficacy and safety of medical ozone (O_3_) delivered in oil suspension applications for the treatment of osteonecrosis of the jaw in patients with bone metastases treated with bisphosphonates: Preliminary results of a phase I‐II study. Oral Oncology, 47, 185–190. 10.1016/j.oraloncology.2011.01.002 21310650

[cre2775-bib-0025] Ruggiero, S. L. , Dodson, T. B. , Aghaloo, T. , Carlson, E. R. , Ward, B. B. , & Kademani, D. (2022). American Association of Oral and Maxillofacial Surgeons' Position Paper on Medication‐Related Osteonecrosis of the Jaws—2022 Update. Journal of Oral and Maxillofacial Surgery, 80, 920–943. 10.1016/j.joms.2022.02.008 35300956

[cre2775-bib-0026] Ruggiero, S. L. , Dodson, T. B. , Fantasia, J. , Goodday, R. , Aghaloo, T. , Mehrotra, B. , O'Ryan, F. , & American Association of Oral and Maxillofacial Surgeons . (2014). American Association of Oral and Maxillofacial Surgeons position paper on medication‐related osteonecrosis of the jaw—2014 update. Journal of Oral and Maxillofacial Surgery, 72, 1938–1956. 10.1016/j.joms.2014.04.031 25234529

[cre2775-bib-0027] Sabri, H. , Sarkarat, F. , Mortezagholi, B. , & Aghajani, D. (2022). Non‐surgical management of oro‐antral communication using platelet‐rich fibrin: A review of the literature. Oral Surgery, 15, 455–464.

[cre2775-bib-0029] Sarkarat, F. , Kalantar Motamedi, M. H. , Jahanbani, J. , Sepehri, D. , Kahali, R. , & Nematollahi, Z. (2014). Platelet‐rich plasma in treatment of zoledronic acid‐induced bisphosphonate‐related osteonecrosis of the jaws. Trauma Monthly, 19, e17196. 10.5812/traumamon.17196 25032151 PMC4080617

[cre2775-bib-0030] Sarkarat, F. , Modarresi, A. , Chiniforush, N. , Yazdanparast, L. , & Rakhshan, V. (2019). Efficacy of photodynamic therapy in minimizing bisphosphonate‐related osteonecrosis of the jaws after dental extraction: A preliminary animal study. Journal of Oral and Maxillofacial Surgery, 77, 307–314. 10.1016/j.joms.2018.09.036 30395823

[cre2775-bib-0031] Sarkarat, F. , Modarresi, A. , Riyahi, A. , Mortazavi, P. , Tabandeh, F. , & Rakhshan, V. (2022). Efficacy of hyaluronic acid, absorbable collagen sponge, and their combination in minimizing bisphosphonate‐related osteonecrosis of the jaws (BRONJ) after dental extraction: A preliminary animal histomorphometric study. Maxillofacial Plastic and Reconstructive Surgery, 44, 8. 10.1186/s40902-022-00337-7 35230522 PMC8888787

[cre2775-bib-0032] Schubert, M. , Klatte, I. , Linek, W. , Müller, B. , Döring, K. , Eckelt, U. , Hemprich, A. , Berger, U. , & Hendricks, J. (2012). The saxon bisphosphonate register—Therapy and prevention of bisphosphonate‐related osteonecrosis of the jaws. Oral Oncology, 48, 349–354. 10.1016/j.oraloncology.2011.11.004 22130456

[cre2775-bib-0033] Scoletta, M. , Arduino, P. G. , Reggio, L. , Dalmasso, P. , & Mozzati, M. (2010). Effect of low‐level laser irradiation on bisphosphonate‐induced osteonecrosis of the jaws: Preliminary results of a prospective study. Photomedicine and Laser Surgery, 28, 179–184. 10.1089/pho.2009.2501 19795990

[cre2775-bib-0034] Urade, M. , Tanaka, N. , Furusawa, K. , Shimada, J. , Shibata, T. , Kirita, T. , Yamamoto, T. , Ikebe, T. , Kitagawa, Y. , & Fukuta, J. (2011). Nationwide survey for bisphosphonate‐related osteonecrosis of the jaws in Japan. Journal of Oral and Maxillofacial Surgery, 69, e364–e371. 10.1016/j.joms.2011.03.051 21782307

[cre2775-bib-0035] Vandenbroucke, J. P. , von Elm, E. , Altman, D. G. , Gøtzsche, P. C. , Mulrow, C. D. , Pocock, S. J. , Poole, C. , Schlesselman, J. J. , Egger, M. , & STROBE Initiative . (2007). Strengthening the reporting of observational studies in epidemiology (STROBE): Explanation and elaboration. PLoS Medicine, 4, e297. 10.1371/journal.pmed.0040297 17941715 PMC2020496

[cre2775-bib-0036] Vandone, A. M. , Donadio, M. , Mozzati, M. , Ardine, M. , Polimeni, M. A. , Beatrice, S. , Ciuffreda, L. , & Scoletta, M. (2012). Impact of dental care in the prevention of bisphosphonate‐associated osteonecrosis of the jaw: A single‐center clinical experience. Annals of Oncology, 23, 193–200. 10.1093/annonc/mdr039 21427065

[cre2775-bib-0037] Vescovi, P. , Merigo, E. , Meleti, M. , Fornaini, C. , Nammour, S. , & Manfredi, M. (2007). Nd:YAG laser biostimulation of bisphosphonate‐associated necrosis of the jawbone with and without surgical treatment. The British Journal of Oral & Maxillofacial Surgery, 45, 628–632. 10.1016/j.bjoms.2007.03.016 17524535

[cre2775-bib-0038] Weiss, H. M. , Pfaar, U. , Schweitzer, A. , Wiegand, H. , Skerjanec, A. , & Schran, H. (2008). Biodistribution and plasma protein binding of zoledronic acid. Drug Metabolism and Disposition, 36, 2043–2049. 10.1124/dmd.108.021071 18625688

[cre2775-bib-0039] Wysowski, D. K. , & Greene, P. (2013). Trends in osteoporosis treatment with oral and intravenous bisphosphonates in the United States, 2002‐2012. Bone, 57, 423–428. 10.1016/j.bone.2013.09.008 24063946

[cre2775-bib-0040] Yüce, M. O. , Adalı, E. , & Işık, G. (2021). The effect of concentrated growth factor (CGF) in the surgical treatment of medication‐related osteonecrosis of the jaw (MRONJ) in osteoporosis patients: A randomized controlled study. Clinical Oral Investigations, 25, 4529–4541. 10.1007/s00784-020-03766-8 33392802

